# Analysis of *Bifidobacterium animalis* subsp. *lactis* BB-12 ^®^ and *Lactobacillus rhamnosus* GG on underweight and malabsorption in premature infants

**DOI:** 10.1590/1806-9282.20230636

**Published:** 2024-02-23

**Authors:** Meng Xing, Xuran Li, Yinzhu Zhang

**Affiliations:** 1 Beijing United Family Hospital, Department of Pediatrics - Beijing, China.; 2 Beijing United Family Jingbei Women and Children’s Hospital, Department of Pediatrics - Beijing, China.

**Keywords:** Bifidobacterium, Lactobacillus rhamnosus, Preterm, Infants

## Abstract

**OBJECTIVE:**

This study aimed to explore and analyze the therapeutic effect of the combination of *Bifidobacterium animalis* subsp. *lactis* BB-12^®^ and *Lactobacillus rhamnosus* GG on underweight and malabsorption in premature infants.

**METHODS:**

This is a retrospective study. The clinical data of 68 premature infants admitted to Beijing United Family Hospital (Private Secondary Comprehensive Hospital, Chaoyang District, Beijing, China) from January 2016 to January 2022 were analyzed retrospectively. Preterm infants less than 37 weeks of gestational age admitted to the neonatal intensive care unit were included in the study. Patients with intestinal malformations, necrotizing enterocolitis, etc., who require long-term fasting were excluded. A telephone follow-up was performed 3-6 months after discharge. They were classified as treatment groups A and B according to the treatment plan. The treatment group A included parenteral nutrition, enteral nutrition, etc. In treatment group B, based on treatment group A, the premature infants were treated with *Bifidobacterium animalis* subsp. *lactis* BB-12^®^ and *Lactobacillus rhamnosus* GG. The time to regain birthweight and the weight on day 30 were compared between the two groups, as was the duration of transition from parenteral nutrition to total enteral nutrition.

**RESULTS:**

The time of weight regain birthweight in group B was shorter than that in group A (t=-2.560; t=-4.287; p<0.05). The increase of weight on day 30 in group B was significantly higher than that in group A (t=2.591; t=2.651; p<0.05). The time from parenteral nutrition to total enteral nutrition in group B was shorter than that in group A (z=-2.145; z=-2.236; p<0.05).

**CONCLUSION:**

In the treatment of premature infants, the combination of *Bifidobacterium animalis* subsp. *lactis* BB-12^®^ and *Lactobacillus rhamnosus* GG can have a better therapeutic effect on the underweight and malabsorption of premature infants, and this treatment method can be popularized in clinics.

## INTRODUCTION

Premature infants, born at less than 37 weeks of gestation, have underdeveloped immune systems and face challenges in gastrointestinal feeding due to slow gastrointestinal tract development. Additionally, related diseases and antibiotic treatment can further delay the establishment of healthy intestinal flora. This negatively impacts nutrient absorption and immune system development, potentially leading to complications like necrotizing enterocolitis (NEC). Probiotics have shown promise in improving feeding tolerance, reducing inflammation, altering intestinal flora, and influencing metabolic mechanisms^1^. However, the mechanism of action, efficacy, and safety of probiotic treatment in premature infants are still under investigation^2^ ^,^ ^3^.

Intestinal microflora, an essential component of the intestinal system, plays a crucial role in the intestinal barrier, digestion and absorption, nutrient metabolism, and immune function^4^. Oral probiotics have been found to enhance gastrointestinal function and lower the incidence of NEC and sepsis in premature infants^1^ ^,^ ^5^ ^,^ ^6^. Nevertheless, the exact mechanisms of action, effectiveness, and safety of probiotic treatment remain uncertain, and there is a lack of clinical studies focusing on premature infants. *Bifidobacterium animalis* subsp. *lactis* BB-12^®^ is involved in host digestion, nutrition, metabolism, absorption, immunity, and anti-infection processes. It helps prevent the invasion of pathogenic bacteria or viruses in the intestinal mucosa, promotes intestinal cell maturation, and facilitates newborn development. *Lactobacillus rhamnosus* GG, a commonly used probiotic, exhibits tolerance to bile and gastric acid, reduces NEC, and improves outcomes in premature infants^7^. However, its impact on intestinal flora and immunity in premature infants is not well understood. In this study, we aimed to investigate the therapeutic efficacy of a combination of *Bifidobacterium animalis* subsp. *lactis* BB-12^®^ and *Lactobacillus rhamnosus* GG in the treatment of underweight and malabsorption in premature infants. The detailed findings are provided below.

## METHODS

### Subjects

**Figure 1.**
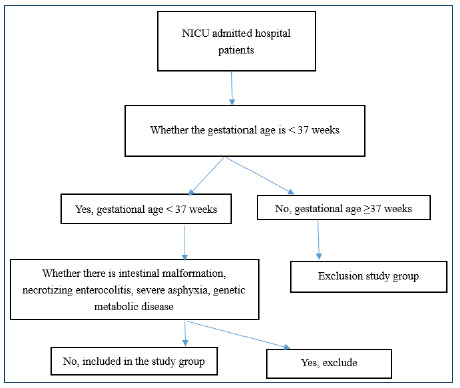
Flow chart.

This is a retrospective study. Participants in the study were 68 preterm infants with gestational age less than 37 weeks admitted to Beijing United Family Hospital (Private Secondary Comprehensive Hospital, Chaoyang District, Beijing, China) between January 2016 and January 2022. Preterm infants less than 37 weeks of gestational age admitted to the neonatal intensive care unit (NICU) were included in the study. The patients were classified as treatment groups A and B according to different treatment methods. Exclusion criteria were as follows: severe asphyxia and infection at birth, congenital malformation of the digestive tract, congenital immunodeficiency, and various inherited metabolic diseases; inability to tolerate gastrointestinal administration; giving up treatment during hospitalization for various reasons; poor compliance, not having follow-up time; use of antibiotics during the study; after discharge without permission stop taking or take other probiotics; and could not complete the study for various reasons. Due to the small NICU scale in our hospital, no patients requiring long-term fasting such as intestinal malformations and NEC, and the one-to-one nursing between nurses and patients in our hospital, nosocomial infections rarely occur, so no patients have been excluded. The specific process is shown in Figure 1.

This study was approved by the Ethics Committee of our hospital (2023-03-006-k06) and obtained the written informed consent of all patients’ legal guardians.

### Data collection

Preterm infants were classified according to gestational age: (1) very preterm infants: preterm infants with gestational age less than 28 weeks; (2) very preterm infants: preterm infants with a gestational age of 28-32 weeks; and (3) premature infants: premature infants with a gestational age of 32-37 weeks. Preterm infants with a gestational age of less than 28 weeks are more immature than preterm infants of other gestational ages. Feeding intolerance (FI) and even NEC are more likely to occur during the feeding process. Before the implementation of enteral nutrition for premature infants, it is necessary to comprehensively evaluate their general condition (gestational age and birth weight: normal range is determined based on gestational age; heart rate: 120-160 beats per minute; respiratory rate: 30-60 breaths per minute; body temperature: 36.5-37.5°C; whether there are obvious breathing difficulties, cardiovascular problems, etc.), physical examination (skin condition, whether jaundice, skin lesions, etc., abdominal distension, tenderness, etc.), laboratory monitoring (hemoglobin level: between 13 and 20 g/dL; white blood cell count: 5,000-15,000/µL; blood sodium level: 135-145 mmol/L; blood potassium level: 3.5-5.0 mmol/L; blood calcium level: 8.5-10.5 mg/dL; blood phosphorus level: 2.5-4.5 mg/dL; indicators of liver and kidney function; inflammatory markers such as C-reactive proteins and cytokines), imaging examination (chest X-ray: lung condition and whether there are any pulmonary issues based on the imaging results), medical history complications (whether preterm infants have other underlying health problems, such as heart disease and neurological problems, whether there are complications such as infection, intestinal necrosis, and intestinal obstruction), and pipeline placement (insertion of an enteral nutrition tube: checking whether the cannula is correctly placed without displacement or air leakage), and whether there are serious contraindications according to the evaluation results should be observed. If so, it is necessary to suspend or stop the enteral nutrition treatment plan^8^.

### Grouping and treatment methods

In group A, premature infants with gestational age less than 28 weeks or weight less than 2 kg were put into the neonatal incubator in time, and the other premature infants were put into the neonatal radiation heater for close observation^9^. For preterm infants without contraindications to enteral feeding, feeding was performed within 12-48 h, with intermittent feeding every 2-3 h. Depending on the condition of premature infants, enteral feeding was increased gradually, combined with the infant’s tolerance, if the daily intake of calories should be insufficient by parenteral nutrition. Premature infants need to be given adequate energy supply after birth, through enteral nutrition, parenteral nutrition, or enteral and parenteral combined energy support, from the first day of 50 kcal/kg/day to the third day of 80 kcal/kg/day, gradually increasing to 110-150 kcal/kg/day energy intake^8^ ^,^ ^10^.

Based on group A, group B was treated with Nemans, 1 g, once a day. For premature infants without feeding contraindications, Nemans was mixed with breast milk or milk powder to feed premature infants once a day. For newborns using antibiotics, the interval with antibiotics is more than 2 h before feeding.

The manufacturer of Nemans is PharmTech (HongKong) Ltd. It is produced in Hong Kong, China, and is sold normally in mainland China. The main ingredients are *Bifidobacterium animalis* BB-12 and *Lactobacillus rhamnosus* LGG. The product is packaged in 1 g/bag, and each bag (1 g) contains at least 4.5 billion viable probiotics.

### Observation indicators

The observational indexes in this study were the time when the weight returned to the birth weight, the weight on day 30, and the duration from parenteral nutrition to total enteral nutrition. The time when the weight returned to the birth weight is defined as the period it takes for the infant’s weight to reach the same weight as at the time of birth. The weight on day 30 is defined as the infant’s weight on the 30th day after birth. The duration from parenteral nutrition to total enteral nutrition is defined as the length of time it takes for the infant’s nutrition to transition from being received intravenously to being fully obtained through the digestive tract.

### Statistical methods

The SPSS 26.0 software was used to analyze the data. Comparisons between two groups with continuous data conforming to the homogeneity of variance of normal distribution were performed using a parametric test (independent-samples t-test), presented as mean±standard deviation, and comparisons between two groups with non-normal distribution data were performed using a Mann-Whitney U test - median and quartile [m (QR)] were used, and categorical data were counted using the chi-square test and frequency (rate). A two-sided test showed a significant difference (p<0.05).

## RESULTS

### General data analysis

A total of 66 patients (33 in each group) were enrolled in this study. In group A, the ratio of males to females was 21:12, the average gestational age was 31.77 weeks, and the average birth weight was 1671.36 g. In group B, the ratio of males to females was 17:16, the average gestational age was 32.85 weeks, and the average birth weight was 1831.06 g. There were no significant differences in sex, gestational age, birth weight, or mode of delivery between the two groups (p>0.05), as shown in Table 1.

**Table 1.** General data analysis (case).

Indicators	Group A (n=33)	Group B (n=33)	t/X^2^ value	p-value
Sex			0.992	0.319
Male	21 (63.6%)	17 (51.5%)		
Female	12 (36.4%)	16 (48.5%)		
Weeks of gestation	31.77	32.85	1.455	0.151
Weight (g)	1671.36	1831.06	1.271	0.208

The treatment group A included parenteral nutrition, enteral nutrition, etc., treatment group B based on treatment group A, the premature infants were treated with *Bifidobacterium animalis* subsp. *lactis* BB-12^®^ and *Lactobacillus rhamnosus* GG.

### Comparative analysis of each index in different gestational age

This study compared the two groups of premature infants over 28 weeks of each index analysis. The time to return to birth weight in group B was significantly shorter than that in group A (t=-2.560, p=0.013), and the difference in weight on day 30 in group B was significantly higher than that in group A (t=2.591, p=0.012). The duration of conversion from parenteral nutrition to total enteral nutrition in group B was significantly shorter than that in group A (z=-2.145, p=0.032), as shown in Table 2.

**Table 2.** Comparison and analysis of the indexes of premature infants above 28 weeks.

Indicators	Group A (n=30)	Group B (n=30)	t/z value	p-value
Time required to return to birth weight (days)	7.79	5.93	-2.560	0.013
Duration of conversion from parenteral nutrition to total enteral nutrition (days)	16.50	8.0	-2.145	0.032
Weight difference (g)	696.96	895.0	2.591	0.012

The treatment group A included parenteral nutrition, enteral nutrition, etc., treatment group B based on treatment group A, the premature infants were treated with *Bifidobacterium animalis* subsp. *lactis* BB-12^®^ and *Lactobacillus rhamnosus* GG.

In addition, this study compared the two groups of premature infants under 28 weeks of gestational age for each index analysis. The time to return to birth weight in group B was significantly shorter than that in group A (t=-4.287, p=0.005), and the difference of birth weight in group B was significantly higher than that in group A (t=2.651, p=0.038). The duration of conversion from parenteral nutrition to total enteral nutrition in group B was significantly shorter than that in group A (z=-2.236, p=0.036), as shown in Table 3.

**Table 3.** Comparison and analysis of the indexes of premature infants below week 28.

Indicators	Whether or not to add probiotics		t/z-value	p-value
	Yes (3 cases)	No (3 cases)		
Time required to return to birth weight (days)	7.0	9.80	-4.287	0.005
Duration of conversion from parenteral nutrition to total parenteral nutrition (days)	50.0	63.0	-2.236	0.036
Weight difference (g)	728.33	443.0	2.651	0.038

The treatment group A included parenteral nutrition, enteral nutrition, etc., treatment group B based on treatment group A, the premature infants were treated with *Bifidobacterium animalis* subsp. *lactis* BB-12^®^ and *Lactobacillus rhamnosus* GG.

## DISCUSSION

In this study, we examined the effect of supplementation with *Lactobacillus rhamnosus* GG and *Bifidobacterium animalis* subsp. *lactis* BB-12^®^ on the return of premature weight to birth weight and the weight on day 30, and the effect of the duration of conversion from parenteral nutrition to total parenteral nutrition.

More than 10,000 premature infants worldwide have been randomized controlled trials for probiotics, suggesting that probiotics can generally reduce NEC, sepsis, and mortality^11^ ^,^ ^12^. However, the answers to clinical questions about which strain to use, the dose, and the timing of supplementation are not clear^13^. On the contrary, a growing number of commercial products containing probiotics are sometimes of poor quality^14^.

*Bifidobacterium* is a common component of the inherent microbiota in the human gut. *Bifidobacterium animalis* subsp*. lactis* BB-12^®^ (BB-12^®^), which is the world’s most documented probiotic *Bifidobacterium*, originates from Chr. Hansen’s collection of dairy cultures, has high stability in foods, and is available as freeze-dried powders. Studies have shown that BB-12 supplementation in preterm infants can effectively promote the proliferation of bifidobacteria in the intestine and inhibit the harmful bacteria *Enterobacter* and *Clostridium*, thus effectively promoting the balance of intestinal flora^15^. BB-12 has a great effect on human health; it can regulate intestinal flora, improve immune function, reduce infection, and improve resistance, so it is often used in infant formula milk powder, food additives, and yogurt^16^ ^,^ ^17^ ^,^ ^18^. Studies have shown that BB-12 has significant clinical efficacy against FI in neonates and diarrhea in infants^19^ ^,^ ^20^. The survival of BB-12 in the gastrointestinal tract has been demonstrated, and BB-2 has been shown to support a healthy gastrointestinal microbiota^21^. In addition, BB-12 has been shown to improve intestinal function, protect against diarrhea, and reduce the side effects of antibiotic therapy. In terms of immune function, clinical studies have shown that BB-12 enhances the body’s resistance to common respiratory infections and reduces the incidence of acute respiratory infections^22^.

*In vivo* studies show that LGG has good adhesion and colonization ability in the human gastrointestinal tract^23^. The possible mechanisms involved in its protective effects on the gastrointestinal tract include enhancement of intestinal barrier function and adhesion to the intestinal mucosa, inhibition of pathogen adhesion, competitive rejection of pathogenic microorganisms, production of antibiotic-like substances, and regulation of the body’s immunity and system. NEC is the most common severe acquired disease in premature infants, characterized by intestinal wall necrosis of different lengths and depths. Intestinal perforation occurs in one-third of affected infants^2^ ^,^ ^3^. The European Society of Pediatric Gastroenterology, Liver Disease and Nutrition (ESPGHAN) probiotics, Prebiotics and Nutrition Committee Working Group published a systematic review and network meta-analysis of the randomized controlled trials in 2018. The report analyzed data on the use of probiotics in preterm infants, outcomes regarding mortality, NEC, delayed sepsis (LOS), or time to complete enteral feeding. There were 51 randomized controlled trials, including 11,231 premature births. Seven treatments reduced the incidence of NEC, two treatments reduced the incidence of NEC and LOS, and three treatments reduced the time to complete enteral feeding. LGG was used in seven trials^24^.

Due to the low number of premature infants under 28 weeks (six cases), in future studies we will include more premature infants. The small sample size and the geographical limitations of the subjects will cause some bias in the conclusion of this study. In addition, when we analyzed the NICU data in our hospital, we found that there were two cases of neonatal sepsis in these two groups; the incidence was lower than that reported in the previous literature, and the infection rate is low. We will do further analysis to include more samples.

In conclusion, the treatment of premature infants with the combination of *Bifidobacterium animalis* subsp*. lactis* BB-12^®^ and *Lactobacillus rhamnosus* GG can obtain a better therapeutic effect on underweight and malabsorption. This study provides a new idea for the clinical treatment and nursing of premature infants, as well as the relevant database.

## Data Availability

The datasets used or analyzed during the current study are available from the corresponding author on reasonable request.
